# Gut dysmotility in children with neurological impairment: the nutritional management

**DOI:** 10.3389/fneur.2023.1200101

**Published:** 2023-05-05

**Authors:** Antonio Corsello, Lorenzo Scatigno, Annalisa Govoni, Gianvincenzo Zuccotti, Frédéric Gottrand, Claudio Romano, Elvira Verduci

**Affiliations:** ^1^Department of Pediatrics, Vittore Buzzi Children’s Hospital, University of Milan, Milan, Italy; ^2^Department of Biomedical and Clinical Sciences L. Sacco, University of Milan, Milan, Italy; ^3^Department of Pediatric Gastroenterology, Hepatology, and Nutrition, CHU Lille, University of Lille, Lille, France; ^4^Pediatric Gastroenterology and Cystic Fibrosis Unit, Department of Human Pathology in Adulthood and Childhood "G. Barresi", University of Messina, Messina, Italy; ^5^Department of Health Science, University of Milan, Milan, Italy

**Keywords:** gastrointestinal dysmotility, gut motility disorders, pediatric neurological impairment, neurometabolic diseases, nutritional management, enteral feeding, cerebral palsy, gut-brain axis

## Abstract

Intestinal motility disorders represent a frequent problem in children with neurological impairment. These conditions are characterized by abnormal movements of the gut, which can result in symptoms such as constipation, diarrhea, reflux, and vomiting. The underlying mechanisms leading to dysmotility are various, and the clinical manifestations are often nonspecific. Nutritional management is an important aspect of care for children with gut dysmotility, as it can help to improve their quality of life. Oral feeding, when safe and in the absence of risk of ingestion or severe dysphagia, should always be encouraged. When oral nutrition is insufficient or potentially harmful, it is necessary to switch to an enteral by tube or parenteral nutrition before the onset of malnutrition. In most cases, children with severe gut dysmotility may require feeding via a permanent gastrostomy tube to ensure adequate nutrition and hydration. Drugs may be necessary to help manage gut dysmotility, such as laxatives, anticholinergics and prokinetic agents. Nutritional management of patients with neurological impairment often requires an individualized care plan to optimize growth and nutrition and to improve overall health outcomes. This review tries to sum up most significant neurogenetic and neurometabolic disorders associated with gut dysmotility that may require a specific multidisciplinary care, identifying a proposal of nutritional and medical management.

## 1. Introduction

Intestinal motility disorders are a group of conditions that have become increasingly prevalent in recent years, and which have a significant impact on the quality of life of children (1). Although most gastrointestinal (GI) motility symptoms do not stem from an underlying organic disease and will resolve on their own, undiagnosed GI dysfunction can interfere with proper growth and nutrition, particularly in infants and young children. In severe cases, it is essential to identify such dysfunction as soon as possible (2).

Pediatric patients with neurological impairment (NI), whether congenital or acquired, and who have comorbidities at different levels of the GI tract, represent a complex issue requiring multidisciplinary, long-term follow-up (3). This is especially true in the case of neurometabolic diseases, a heterogenous group of inherited disorders characterized by alterations of specific aspects of cellular metabolism (4). These patients are at an elevated risk of malnutrition, which can have serious consequences if not properly addressed. Therefore, it is crucial to identify and treat any GI dysfunction in this population promptly.

It is now known that there is a strong bidirectional communication between the central nervous system (CNS) and the enteric system, which is described under the term gut-brain axis (5). In fact, neural communication has important integrative roles in gut function. The anterior insular cortex (referred to as the visceral cortex), prefrontal and sensory/motor regions, cingulate gyrus and limbic regions have been found to be involved in the integration of neural information in the GI tract (6). The gut-brain axis modulates GI motility, gastric secretion, blood flow, gut barrier integrity, immune response, and visceral sensations (7, 8). In addition, patients with functional GI disorders show structural and functional information processing abnormalities of the visceral and vasovagal motor system (9–11).

In addition to the complex neuronal interaction, the gut-brain axis is also characterized by a complex system of hormonal and biohumoral interactions, such as an active modulation of the intestinal microbiota (5). The gut microbiota is a complex ecosystem of microorganisms that play an essential role in various physiological functions, including digestion, immune function, and metabolism (12). The dysbiosis of the gut microbiota can lead to alterations in the gut-brain axis, contributing to the development and exacerbation of neurological symptoms (7, 13). Resident microbial organisms can lead to increased intestinal epithelial permeability and modulation of the immune response of the host organism (13). Moreover, changes in gut microbiota can modulate neurotransmitter synthesis and consumption, alter perception of gut stimuli, and modulate various GI functions (14, 15). Disorders of the gut barrier and changes in emotional state induced by the gut microbiota alter the interaction of the gut-brain axis. Indeed, if the nervous system regulates normal gut functions, the gut system can also modulate brain function (5). Because of this close relationship between the brain and gut, a rationale for how brain pathologies can affect the functioning of the GI system is needed. Diet then plays a critical role in shaping the gut microbiota and maintaining microbial diversity, and thus, nutritional interventions aimed at promoting a diverse and healthy microbiota may have important therapeutic potential in the management of these patients (16).

The aim of this review is to summarize the recent evidence on congenital neurological disorders, both acquired and genetic, and neurometabolic syndromes involving GI motility to identify some patterns that might benefit from different specific approaches and perspectives regarding nutritional therapy. To further investigate the relationship between the gut-brain axis and neurological disorders, we aimed to evaluate dysmotility in two models of disability: acquired stable model of cerebral palsy (CP) and one associated with neurogenetic/neurometabolic pathologies which are associated with degenerative models. The dysfunction of the gut-brain axis in these models is an area of interest as it can provide insights into potential targeted interventions for improving GI motility and nutritional therapy. This review will provide a comprehensive understanding of the current evidence and gaps in knowledge in the field of GI motility in patients with severe NI.

## 2. Materials and methods

This narrative review includes an up-to-date summary of the current literature on gut dysmotility in patients with severe NI, and the nutritional management of these conditions. A comprehensive search of Pubmed/Medline, Embase, and Web of Science databases was conducted by two independent authors to identify the most relevant articles, including original papers, meta-analyses, and reviews. The search strategy included following keywords (alone or in combination): gut dysmotility, gastrointestinal dysmotility, GI disorders, pediatric neurological impairment, neurometabolic disorders, nutritional management, mitochondrial diseases, gut-brain axis, growth, malnutrition. We conducted a literature review to investigate the relationship between GI symptoms and the most frequent causes of neurologic impairment in pediatric patients. Our review focused specifically on genetic diseases and included an assessment of the nutritional status of affected patients, as well as the clinical management of their symptoms. To improve our search results, we also consulted reference lists from most relevant publications. Only papers published in English up to January 2023 were included in this review.

## 3. Gut dysmotility: a brief overview

The definition of intestinal dysmotility includes various disorders, all characterized by impaired muscle activity of the GI tract with altered peristalsis (1). Dysmotility could then lead to eventual bolus retention, fluid and stool entrapment (e.g., pseudo-obstruction), symptoms associated with hyperperistalsis (e.g., diarrhea) or other non-specific disorders such as nausea and malabsorption (3). A subsequent unusually slow, fast or irregular transit could then cause intestinal dysmotility disorders or be the cause itself (2).

Considering the pediatric population, dysmotility disorders can range from transient benign conditions related to an acute etiology or local inflammation to insidious progressive chronic disorders that can even take years to diagnose (17). Furthermore, technical and functional GI assessment in children is more difficult than in adults due to the ambiguous interpretation of the results and the frequent lack of child-specific information (17).

Dysmotility can then be considered as a heterogeneous group of disorders that could identify both a peripheral cause involving impairment of a particular muscle tract or nerve, and a central disorder related to a neurological, hormonal or genetic dysfunction (18). The control of the longitudinal and circular smooth muscles of the intestine is determined by many factors, such as local hormones and neurotransmitters produced at various levels, at both its enteric and central localization, with a wide and sometimes conflicting bowel activity (19). For all these reasons, various specific causes of GI dysmotility can be divided into functional or organic causes.

Although the mechanisms leading to GI dysmotility in children with intestinal failure, short bowel syndrome, anatomical malformations, or primary neuromuscular disease are relatively well understood, the pathophysiology of symptoms in children under 2 years of age is not always clear. In some cases, symptoms may be absent or misdiagnosed, leading to severe complications such as bacterial overgrowth, microbial dysbiosis, and chronic malabsorption, which can significantly impact patients’ quality of life in the long term (18). Furthermore, the increased survival time of many CNS diseases resulting from advances in pharmacological approaches presents a significant challenge that requires deeper knowledge about motility disorders. Most GI dysmotility disorders are characterized by frequent and transient conditions such as constipation and mild delayed gastric emptying, which usually do not require changes in diet (20).

Moreover, it has to be highlighted the importance of the gut microbiota in maintaining gastrointestinal health and the development of neurological conditions. Research has demonstrated that commensal bacteria can influence gut motility through the release of substances mediated by intestinal neuroendocrine factors and the immune response (21). Indeed, dysmotility of the gastrointestinal tract can lead to alterations in the composition of the gut microbiota, which can further exacerbate the underlying neurological symptoms (22). As a proof of this, microbiota can influence gastric and esophageal motility in addition to colonic motility (23, 24). Given this premise, it is easy to understand how modulation of the microbiota by biotics can indirectly affect gut motility.

Assessment of colonic neuromuscular function can be carried out using various methods, including radiopaque markers, colonic scintigraphy and manometry, wireless motility capsule, and electromagnetic capsule tracking systems. The choice of assessment method should be tailored to the clinical context and the severity of the GI dysmotility, considering that conflicting results and significant differences in the use of different methods between centers are still common (25). In the following sections, we will explore when clinicians should recognize an increased risk of developing GI dysmotility and what nutritional approaches may benefit patients.

**Figure 1:**
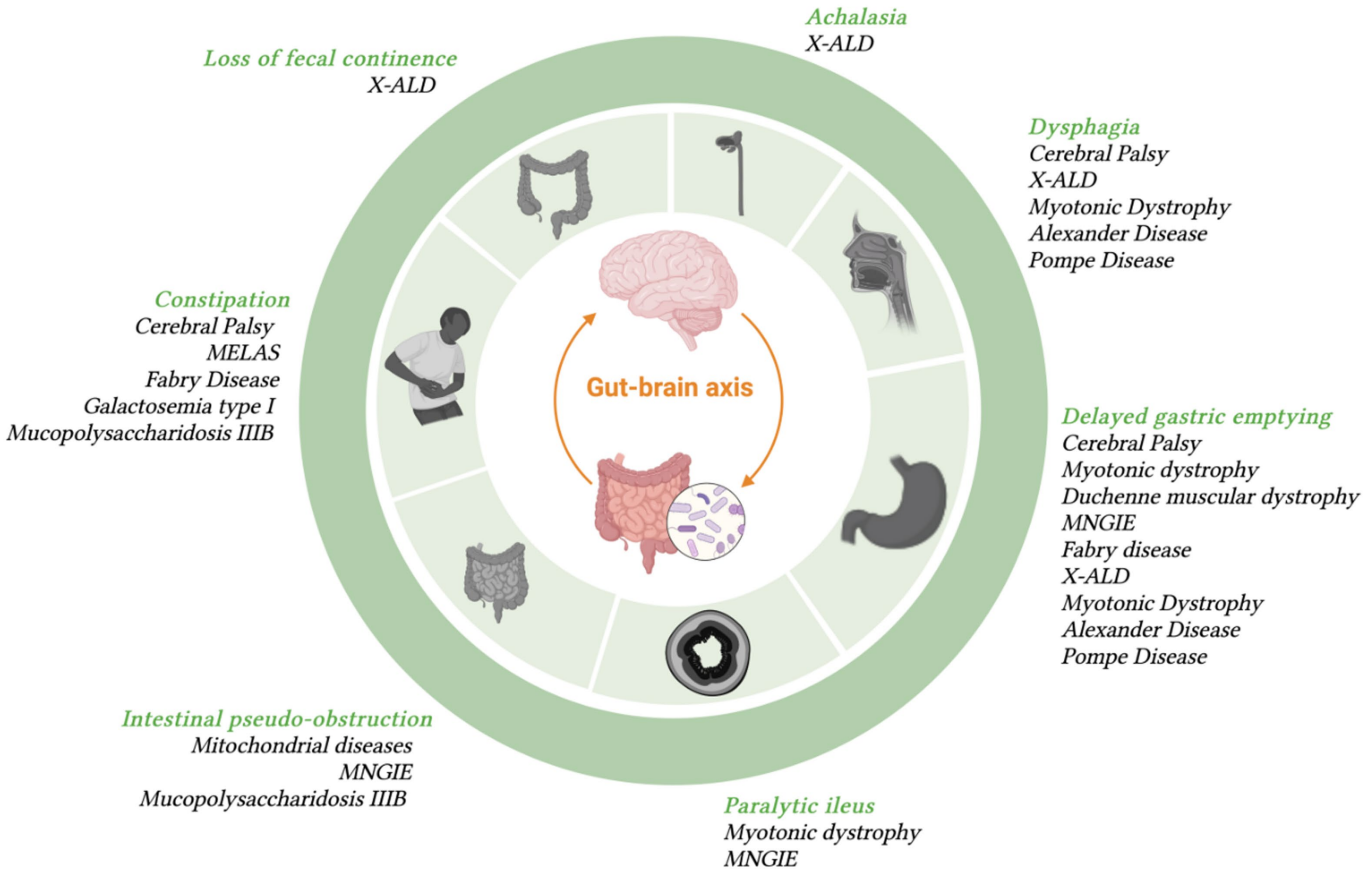
Most frequent GI symptoms occurring in specific diseases with neurologic symptoms.

Figure 1 summarizes some examples of GI symptoms and disorders that have been commonly reported in different type of patients with NI that will described below.

## 4. GI dysmotility and cerebral palsy

Among the pediatric neurological diseases, cerebral palsy (CP) is one of the most frequent with an estimated prevalence of 2.11 per 1,000 live births (26). CP is characterized by disturbances in movement and posture development, leading to limitations in task performance, resulting from nonprogressive damage to the CNS during fetal or perinatal neural development (27).

Prenatal events such as congenital infections and asphyxia account for about 80% of CP causes (28). Perinatal problems such as peripartum asphyxia, uterine rupture, low birth weight, acute maternal viral infections, trauma, hypoxia, or infections such as meningitis are also potential causes of CP (29). Due to the heterogeneity of severity, ranging from mild motor disability to severe cognitive retardation, the child with CP is a complex patient (27). As the severity of the lesion increases in patients with CP, comorbidities such as gastroesophageal reflux (GER) become more prevalent, with a prevalence ranging between 15 and 77% (30, 31). Delayed gastric emptying related to the underlying neurological damage is the primary cause of this evidence. Hypomobility, enforced bed rest, scoliosis increasing intra-abdominal pressure, and use of anticonvulsants exacerbating nausea and vomiting can also contribute to GER (31).

Swallowing is a complex process controlled by cortical and subcortical pathways, including the brainstem, to avoid dysphagia (32). Damage to the cortex can cause voluntary swallowing disorders, while subcortical or basal ganglion lesions can lead to reflex swallowing abnormalities (33). In patients with CP, problems with voluntary and involuntary swallowing often coexist, indicating extensive damage. However, a study by Spiroglou et al. did not observe a correlation between prolonged gastric emptying time and GER severity in patients with CP (34). Constipation has been reported in patients with CP, with a prevalence ranging from 26 to 74%, mainly due to hypomobility associated with the underlying disease (35). It has also been shown to be associated with central programming of bowel movements (36). Consequently, the patient’s GI manifestations should not be interpreted as indicating a local damage, since proper functioning of the central regulation is necessary for the physiology of the entire GI tract.

## 5. GI dysmotility and genetic disorders

### 5.1. Alexander disease

The classic pattern of neurological disorders is not the only one affecting GI motility. Neurometabolic disorders are rare and complex disorders that result from inborn errors of metabolism that lead to impairment of the brain. Depending on the metabolic processes involved, neurometabolic diseases can be classified according to the tissue predominantly involved and the age of the child at presentation (37). Alexander disease is a cerebral degenerative disorder caused by a mutation in the GFAP gene encoding the main intermediate filament of astrocytes, resulting in progressive neurological and cognitive deterioration (38). There are four distinct forms of pathology: neonatal, infant, adolescent, and adult; they differ according to the clinical spectrum and neurological manifestations (39). These patients then require nutritional support because of weak sucking and severe swallowing difficulties (40). These patients often suffer from GERD in addition to the risk of malnutrition and cachexia associated with their neurological condition (41, 42). It has been observed that dysbiosis, or imbalance of gut microbiota, may contribute to the development of GERD by promoting the translocation of fermenting bacteria from the colon to the small intestine (43). This process can lead to the production of fermentative gases that exit from above, contributing to reflux symptoms. While increasing bicarbonate production may be a potential corrective nutritional intervention, other strategies aimed at modulating gut microbiota, such as probiotics or prebiotics, may also be effective in improving GERD symptoms (44, 45). Moreover, dietary interventions that target specific nutrients, such as fiber, may also improve gut health and reduce GERD symptoms (46).

### 5.2. X-linked adrenoleukodystrophy

X-linked adrenoleukodystrophy (X-ALD) is a neurometabolic disorder in which the brain, spinal cord, adrenal glands and testicles are the most affected organs (47). X-ALD represents the most prevalent peroxisomal disorder, originating from mutations occurring in the ABCD1 gene, which encodes the peroxisomal membrane protein ALDP, which plays a critical role in the transmembrane transport of very long-chain fatty acids (48). It is characterized by progressive symmetrical demyelination of the white matter (49). This condition determines progressive neurological damage leading to loss of swallowing ability and fecal continence (50). As in CP, in X-ALD there is no direct involvement of the GI system and consequently the GI problems are related to hypomobility and a loss of central co-regulation and control (47). Therefore, although CP represents static damage while X-ALD is characterized by dynamic progression, patient management might be similar.

### 5.3. Myotonic dystrophy and Duchenne muscular dystrophy

Myotonic dystrophy is an autosomal dominant genetic disorder. It is caused by unstable expansion of a nucleotide triplet containing cytosine-thymine-guanine located in the 3′ untranslated region of chromosome 19q13.3 (51). Involvement of one of the various tracts of the GI system is common in these patients. Dysphagia, regurgitation, and delayed gastric emptying have been reported for the upper GI and abdominal swelling, diarrhea, or constipation for the lower GI (52). In this case, the pathogenesis of the clinical manifestations is local muscle damage; indeed, at the esophageal level, a decrease in muscle tone was observed that is directly proportional to the extent of GER (53); at the gastric level, the impairment of smooth muscle fibers leads to a slowing of gastric emptying, which can lead to gastroparesis (52). Decreased peristaltic activity in the gut can lead to paralytic ileus or anaerobic bacterial overgrowth, which can lead to osmotic diarrhea as a result of malabsorption (54). Paralytic ileus and bacterial colonization can then lead to serious complications such as toxic megacolon (55).

Duchenne muscular dystrophy (DMD) is a sex-linked recessive disease, whose gene is located on Xp21 (56). The GI manifestations in DMD are principally related to the degeneration of the GI smooth muscle that leads to atrophy (57). Gastric emptying time has been found to be significantly longer in patients with DMD than in healthy controls (57). GI damage is progressive and refractory gastroparesis has been frequently described in end-stage DMD patients (58, 59). This disorder can lead to severe gastric and small bowel dilation, leading to a paralytic ileus that often requires decompression surgery to avoid potentially fatal consequences. Similar complications may also occur in the distal segment of the intestine; in fact, reduced motility was observed not only in the stomach but also in the colon (60, 61).

### 5.4. Spinal muscular atrophy type 1

Spinal muscular atrophy type 1 (SMA1) is an autosomal recessive neuromuscular disease mainly caused by a homozygous mutation or deletion in the SMN1 gene (62). GI symptoms observed in SMA1 patients include constipation and delayed colonic transit, GER, delayed gastric emptying, dysphagia and vomiting (63). These symptoms are caused by a defect in the central nervous system, even if pathogenetic role played by the enteric nervous system has been shown. In addition, there is often a significant loss of lean mass in these patients, and it is not possible to compare them to the general population. Specific growth curves and patterns that should be considered in children with SMA1 have been recently published by De Amicis et al. (64). Nutritional interventions can be used to support patients with SMA1 (65). One such intervention could be the supplementation with omega-3 fatty acids, that could improve mitochondrial function and energy metabolism, leading to improved muscle strength and function in some patients (66, 67).

Since survival rates of patients with SMA1 are significantly increasing thanks to the recent gene therapies, an individualized dietary therapy per each age should always be evaluated in order to provide proper growth and nutritional status. Each patient may have specific nutritional needs and metabolic aspects should be considered, with a subsequent strict nutritional and endocrine surveillance (65). Among these patients feeding can be continuous, discontinuous or an alternative administration of the two. A crucial aspect is deciding the proper moment to introduce an enteral nutrition by tube. Firstly, until an adequate weight for surgery is achieved, the placement of a nasogastric tube may be performed. Temporary enteral feeding should be continued until a definitive stoma can be placed.

### 5.5. Mitochondrial diseases

They represent a group of genetic disorders characterized by defects in oxidative phosphorylation caused by mutations in nuclear or mitochondrial DNA genes encoding structural mitochondrial proteins (68). Mitochondrial diseases have a prevalence of about 12.5 per 100,000 inhabitants (69). Because mitochondria are ubiquitous, the clinical presentation is heterogeneous and multisystemic. Its symptoms may include fatigue, skeletal muscle weakness, growth retardation, blindness, ophthalmoplegia, nystagmus, hearing loss, hypoglycemia, diabetes mellitus, learning disabilities, intellectual disability, neuropsychiatric symptoms, cardiomyopathy. Regarding the GI, the muscular organs of the gastroenteric system are significantly involved, as the muscle necessarily requires mitochondrial metabolism to function, and GER, dysmotility and pseudo-obstruction are often described (18, 70).

Esophageal sphincter dysfunction has been commonly reported in MELAS (mitochondrial encephalopathy, lactic acidosis and stroke-like episodes), a mitochondrial disease (71, 72). GER and achalasia are common disorders described in patients with other mitochondrial diseases (73). Furthermore, GI involvement does not generally end at the esophageal level, and GI motility disorders with delayed intestinal transit and severe constipation are commonly reported (74). A study by Bhardwaj et al. analyzed gastric emptying and intestinal transit time in children with mitochondrial disorders and assessed response to prokinetic therapy (75). They found that about 70% of the enrolled patients had prolonged gastric emptying time and almost half had altered intestinal transit. The administration of prokinetics also did not lead to significant improvements.

In mitochondrial neurogastrointestinal encephalopathy (MNGIE), a rare autosomal recessive metabolic disease resulting from mutations in the TYMP nuclear gene, involvement of the GI tract is prominent, markedly delayed gastric emptying and a megacolon, often requiring urgent surgery, have been described (76). Gastroparesis and intestinal pseudo-obstruction are the extreme consequences of this dysmotility (77, 78). GI complications related to dysmotility are the leading causes of death in patients with MNGIE (79, 80). The lack of activity of thymidine phosphorylase causes the systemic accumulation of substrates of pyrimidine deoxyribonucleosides, which cause changes in the stability of mitochondrial DNA (81–84).

Mitochondrial abnormalities seen in MNGIE might contribute to gut bacterial overgrowth or gut microbiota dislocation and cause manifestations of GI dysmotility in these patients (79, 85). MNGIE is commonly associated with chronic intestinal pseudo-obstruction (CIPO), a syndrome of intestinal obstruction without the presence of anatomical or mechanical obstruction, which can result in severe intestinal motility failure (86). In most patients, CIPO leads to persistent malnutrition, and parenteral nutrition is often used (87). The cause could be interstitial Cajal cells (ICC) deficiency or an abnormal distribution of ICC networks, which have been reported in the small intestine and colon of pediatric patients and adults with intestinal pseudo-obstruction (88–90). Abnormalities in ICC number and structure have also been associated with other GI motility disorders such as gastroparesis (91, 92). Some studies suggest that ICCs act as specialized pacemaker cells of the GI (93, 94). As these cells are rich in mitochondria, they could be a target and consequently their deficiency could be the primary event preceding the muscular and neurogenic changes in the MNGIE gut (95). However, the factors contributing to ICC deficits could be caused by other metabolic disorders and GI dysmotility and require more detailed studies as currently available treatments for MNGIE are insufficient to resolve GI manifestations (79, 91).

It is then necessary to remember that in mitochondrial diseases the CNS is often affected and therefore some GI symptoms could be associated with a local muscle type problem but could have a central etiology; it is for example the case of dysphagia (96). Indeed, dysphagia may be related to central impairment, to peripheral nerve damage, or to smooth muscle cell involvement; So, the stronger the neurological involvement, the more likely it is that the root cause of gut problems is also central.

### 5.6. Fabry disease

Symptoms such as abdominal pain, nausea, vomiting and diarrhea, which are frequently reported, should be considered in patients with Fabry disease (97–99). Fabry disease is a rare multisystem metabolic disease resulting from multiple types of mutations in the GLA gene that cause deficiency of the lysosomal enzyme alpha-galactosidase A, resulting in accumulation of glycosphingolipids, particularly globotriaosylceramide, in the lysosomes leads to (GL3) (98, 100, 101). The accumulation of these glycosphingolipids in cells, mainly in the vascular endothelium, leads to progressive cell death and dysfunction of important organs such as kidneys, heart, brain and skin (97, 100, 102). In children suffering from this disease, there are often symptoms associated with GI dysmotility due to autonomic dysfunction, such as abdominal pain, episodes of nausea and vomiting, bloating, and alternating constipation and diarrhea (100, 103, 104). It has been extensively described that abdominal pain and swelling increase immediately after a meal (97, 104). Dyspepsia and delayed gastric emptying can lead to food refusal and, in the worst case, in combination with other GI symptoms, also negatively affect body weight (99, 104, 105). Dysfunction of the autonomic nervous system responsible for gut motility and affecting GI circulation seems to play a central role alongside tissue inflammation related to GL3 accumulation (104, 106).

The therapy currently used for this disease is enzyme replacement therapy, which consists of administration of the recombinant human enzymes alpha-galactosidase A and Agalsidase beta (98, 101). Studies have shown that both enzyme replacement therapies have a GI effect, reducing abdominal pain and diarrhea (103, 107).

### 5.7. Galactosemia type I

GI symptoms, particularly constipation and nausea, are also present in galactosemia type I (108). Galactosemia type I or classic galactosemia is a rare metabolic disorder occurring in neonates and consisting of a deficiency in galactose-1-phosphate uridylyltransferase (GALT) activity (109). Although identification through neonatal screening and prompt intervention to eliminate galactose from the diet eliminates possible acute or fatal symptoms (109, 110), most families still report GI problems in their children (108), probably due to the two different variants of GALT present in pathology and the various dietary restrictions suggested by physicians (111–113). Kelly et al. have suggested that excluding dairy products from the diet may have a negative impact on the gut microbiota of these children (114, 115), and it has been postulated that they may benefit from probiotic supplementation (108).

Artificial nutrition can help to manage the condition by providing sources of nutrition free from galactose. Specialized formulas that are free from lactose and galactose are available and can be used to meet the nutritional needs of individuals with galactosemia type I (116). Individuals with galactosemia type I may be at risk of nutrient deficiencies due to their restricted diet. It is important to monitor their nutrient intake carefully and supplement as necessary to ensure they are meeting their nutritional needs. Galactose can be found in many foods and ingredients, including some medications and supplements (109). It is important to read food labels carefully and avoid any products that contain galactose or lactose. In some cases, individuals with galactosemia type I may require enzyme replacement therapy or a liver transplant (117).

### 5.8. Pompe disease

Type II glycogen accumulation disease, also known as Pompe disease, is an autosomal recessive disorder caused by alpha-glucosidase acid deficiency and results in abnormal accumulation of glycogen in the heart, skeletal and smooth muscles and in the nervous system (118–120). Since smooth muscle is involved, functional manifestations of the GI tract are directly related to the disorder; the most reported are abdominal pain, difficulty feeding and swallowing, GER, postprandial swelling, early satiety, abdominal discomfort, chronic diarrhea, and increased urgency (121–124). Autopsies of patients with Pompe disease revealed accumulation of glycogen in both the striated muscles of the tongue and the proximal esophagus, causing dysphagia, but also in the smooth muscles of the distal esophagus and small intestine, which could lead to other GI functional symptoms (125, 126). Although some cases of symptom improvement with enzyme replacement therapy (ERT) have been described (123, 124, 127), most patients still suffer from a variety of clinical symptoms, including GI symptoms, over the long term, since ERT appears to be inefficiently administered to target tissues (skeletal and smooth muscle) (118).

### 5.9. Sanfilippo syndrome

GI disorders, including diarrhea and constipation, have been described in patients with mucopolysaccharidosis (MPS) IIIB or Sanfilippo syndrome, a rare autosomal recessive lysosomal accumulation (128). The disease results in progressive severe CNS deterioration with severe neurological, cognitive and behavioral symptoms (129, 130). The United Kingdom Society for Mucopolysaccharidoses and Related Diseases has found that GI problems including constipation, diarrhea, food intolerance, recto-vaginal fistula, malabsorption, intestinal volvulus, intestinal congestion, ulcerative colitis, GI bleeding and pseudo-obstruction are commonly reported in these patients (131). A case of chronic diarrhea in MPS IIIB has also been described in the literature, in which abnormalities were found on intestinal endoscopy, partially overlapping with features of intestinal lymphangiectasia, for which the patient was on a low-fat diet and medium-chain triglyceride supplementation, thereby improving the condition of diarrhea (129). Furthermore, functional decline in children from the second decade of life leads to dysphagia and aspiration problems, necessitating nasogastric or gastrostomy feeding (132, 133). Unfortunately, no treatment has yet been discovered for this pathology, and GI complications, which are often underestimated, resulted in 6% of deaths (131).

## 6. The nutritional management of children with severe NI

The increasing survival rates of neurologically impaired patients, including children with genetic syndromes and neurometabolic disorders, require a focus on nutritional and GI issues. Although physical and mental disabilities are major concerns, the enteric nervous system contains more neurons than the spinal cord, so it is hardly surprising that brain damage can result in impaired GI motility and feeding abilities (134, 135). In recent decades, multidisciplinary nutritional programs have aimed to reduce GI manifestations and improve the nutritional status of children with disabilities. In 2017, the European Society of Gastroenterology, Hepatology and Nutrition (ESPGHAN) published a consensus statement on the diagnosis and management of GI and nutritional complications in children with NI (3). These recommendations aimed to provide consistent guidelines for the management of GI manifestations and nutritional status in neurologically impaired children.

The introduction of newborn screening, targeted feeding patterns, and new therapeutic options have contributed to longer survival and improved intestinal dysmotility in children with inherited metabolic disorders. Early dietary manipulation of patients with enzyme-deficient pathways lowers tissue and plasma concentrations of toxic substrates and provides deficient products. Studies over the years have allowed individualization of dietary patterns according to individual tolerance of the toxic metabolite, stage of development and clinical status.

**Figure 2:**
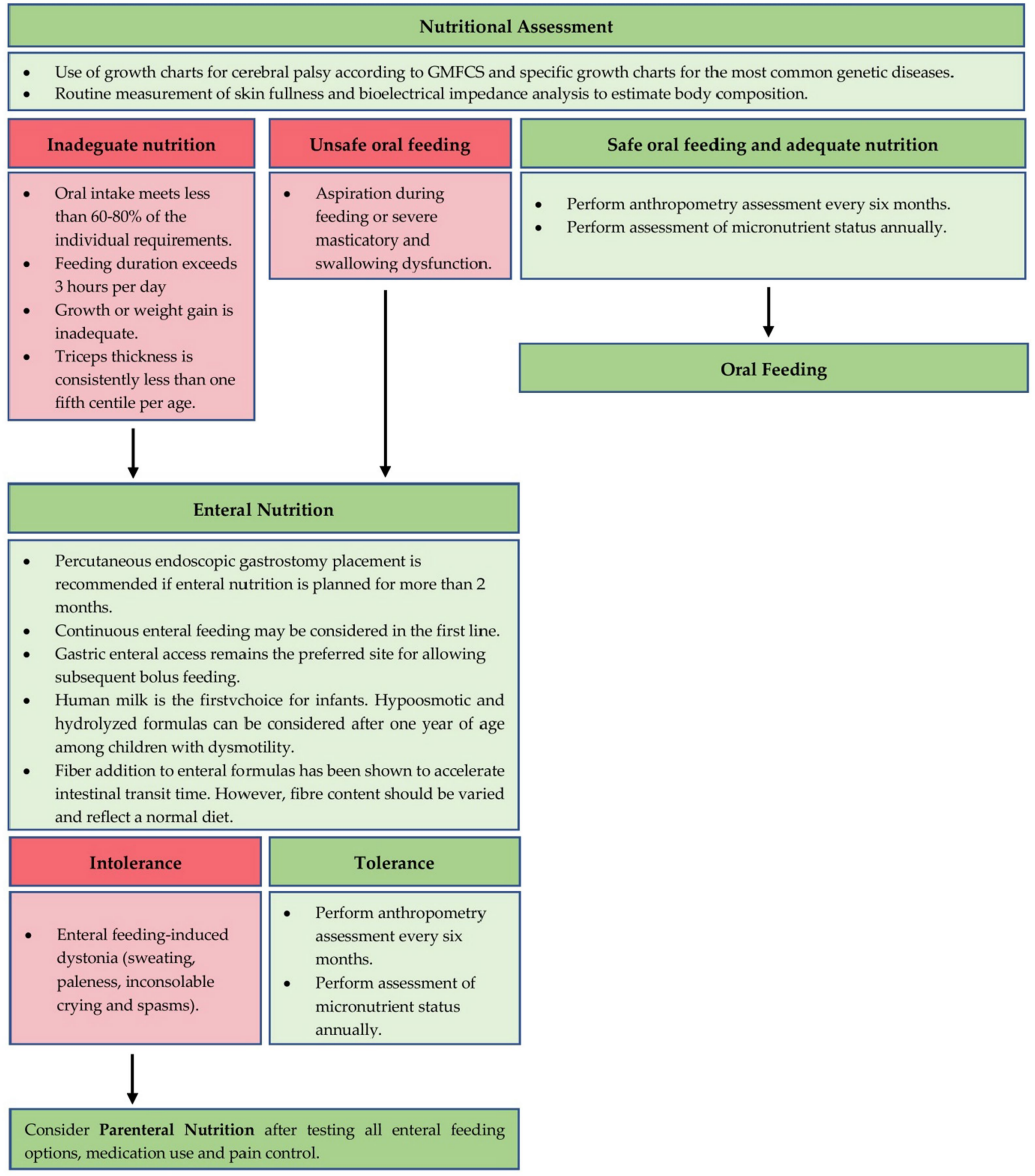
A proposal for nutritional management of GI dysmotility in children with NI.

Figure 2 describes a possible nutritional approach in children with intestinal dysmotility and nutritional impairment.

### 6.1. Nutritional assessment

Nutritional assessment of children with NI is part of multidisciplinary management performed by physicians, dieticians, nurses, speech therapists, physical therapists, psychologists, and occupational therapists. History-taking is the first step and aims to examine the evolution of eating stages and, if present, history of feeding problems since birth. Subsequent assessment of anthropometry is critical for assessing nutritional status and should be performed every 6 months in children with NI (3). Brooks et al. provided CP-specific growth curves stratified by sex, age and functional capacity according to the Gross Motor Function Classification System (GMFCS) (136). For the most common genetic disorders (e.g., Down Syndrome, Turner Syndrome, SMA1, Prader-Willi Syndrome, Rett Syndrome, Noonan Syndrome, Cornelia de Lange Syndrome, Rubinstein-Taybi Syndrome, Duchenne Muscular Dystrophy and Silver-Russell Syndrome) specific growth charts are generally available. However, assessment of height reflects adequate growth and nutritional status but can be challenging in children with malformations and spasticity (137).

Measuring segment length (knee height and tibial length) offers an alternative in children with skeletal deformities where back height cannot be measured (3). Other two measurements commonly used in normally developing children, such as weight-to-age and weight-to-height ratios (body mass index), overlook muscle and fat mass and are poor predictors of body composition in children with NI. Incorrect determination of body mass index could lead to overeating in children with NI, who were found to have a higher percentage of central body fat mass (138). The ESPGHAN position paper recommends routine skinfold thickness measurement and bioelectrical impedance analysis to estimate body composition independent of fat site (3).

Finally, the use of GMFCS growth charts for assessment of nutritional status in children with NI is not recommended as they are descriptive without reference standards (3). There is no clear definition of malnutrition in typically developing children or in children with neurological disorders (139, 140). For clinical practice, one or more of the following warning signs should be used to identify malnutrition in children with NI: physical signs of malnutrition, age-related z-score < 2, triceps plica thickness < 10 percentile for age-sex, mid-adipose muscle area am Upper arm <10 percentile and weight loss or failure to thrive (3).

The use of nutritional reference standards for children with normal development can be considered to estimate the caloric needs of children with NI. In certain clinical situations, such as pressure sores or in children with low calorie needs, it is recommended supplemental protein intake. Micronutrient intake should be in line with dietary reference values for normally developing children (3). The assessment of the micronutrient status (e.g., Vitamin B9, B12, vitamin D, calcium, phosphorus, iron) should be carried out annually. Finally, the use of indirect calorimetry to evaluate the basal metabolism, in addition to dual energy X-ray absorptiometry (DXA) and BMI, may allow examining the nutritional assessment of the child with NI (3, 141).

Nutritional difficulties in children with neurometabolic disorders are often associated with neurological sequelae of the disease. However, there is little evidence describing their frequency or severity (142). Dietary restrictions in congenital neurometabolic disorders can result in deficiencies in fatty acids, essential amino acids, and micronutrients. Close monitoring of nutritional fitness requires close monitoring of growth. Monitoring of weight gain and information on possible long-term consequences of overweight and obesity should be provided (143).

The severity of the main clinical features of dysmotility reflects the ability of a child with NI to adopt and tolerate a regular diet. In addition, the duration of the symptoms can influence the worsening of malnutrition (144). Adjusting nutrition to meet the needs of a neurologically impaired child with GI motility disorders is challenging. Different nutritional approaches include oral nutrition, enteral nutrition, and parenteral nutrition.

### 6.2. Oral nutrition

Maintaining feeding ability is an excellent strategy to increase calorie density in severely malnourished children. Due to the higher energy density of fats per gram (9 kcal/g) compared to proteins and carbohydrates (4 kcal/g), it is recommended to use typical household products such as oils and high-fat spreads in children, and high-calorie formulas in infants (135). Fasting stimulates autophagy through multiple mechanisms related to neurometabolic disorders (145). Abnormalities in mTOR signaling have been reported in Pompe disease after fasting; however, these abnormal signaling pathways were reversed by arginine supplementation (146).

Pharyngeal hypotension associated with generalized hypotension is a common finding in children with neurologically impaired (NI) genetic syndromes. In particular, children with 22q11 deletion syndrome (DiGeorge syndrome, velocardiofacial syndrome, and conotruncal anomaly-facial syndrome) have difficulty increasing the amount of food and changing the consistency of food (147). Therefore, offering small, frequent meals may be a common strategy (148). In children with Prader-Willi syndrome, pharyngeal hypotension leads to poor sucking. Using a nipple with a wider base can help the infant achieve a seal around the nipple and allow intraoral pressure to build up. Supporting the head in a neutral or slightly flexed position while applying intense pressure to the midline of the tongue may improve tongue tone (149).

A variety of esophageal dysmotility disorders can significantly interfere with oral feeding in children with NI. Patients should be encouraged to eat upright and minimize food intake for several hours before bedtime. Other esophageal motility disorders affect only the absorption of solid food. The diet should therefore allow for fluids and mixed foods, as well as fluid intake after each swallow if solid meals are tolerated (144). Children with delayed gastric emptying should eat finely divided food in 5 or 6 small meals per day. The diet should be low in fiber to prevent bezoar formation and low in fat to avoid exacerbating delayed gastric emptying (144). Studies conducted among infants report better gastric emptying with medium-chain triglycerides than long-chain triglycerides (150), while whey hydrolysate formulas have been shown to improve feeding tolerance and reduce gastrointestinal symptoms in some patients, with a faster emptying (151). In contrast, a higher fluid content in the diet should be encouraged as it is better tolerated. Regardless of the consistency of the food offered, the child should sit for 1–2 h after the meal (152). Finally, in children with NI and constipation, increasing fiber and fluid intake may be a useful strategy to improve this symptom (153). Reports of the use of mixed diets in patients with dysmotility have recently emerged, although pediatric data are still limited. Individual studies have shown an improvement in nausea and vomiting due to the higher viscosity of the mixtures (154, 155). A possible positive effect on stool frequency and consistency has been associated with the introduction of various dietary fibers (155). The positive influence of the mixed diet on the diversity and richness of the intestinal microbiome was described, with a significant increase in bacterial diversity and a corresponding decrease in harmful Proteobacteria in the analyzed stool samples (156). However, more research is needed to evaluate the composition and safety of mixed diets in children with dysmotility.

Finally, it has to be stated that completely abandoning oral nutrition in children is a clear indication of a failure in medicine, since the proper maturation of the entire digestive system represent the best solution for ensuring proper nutrient absorption and supply to the body (157). However, in some cases, children with NI may face difficulty in maintaining a full oral nutrition (3). In such cases, it is important to address the root cause of the problem through proper medical intervention and nutritional support.

### 6.3. Enteral feeding by tube

Enteral nutrition is recommended for patients who cannot eat orally for more than 3 h a day, whose oral intake provides less than 60–80% of their individual needs, whose growth or weight gain are insufficient, and whose triceps thickness is consistently below the fifth percentile per age. Aspiration during oral feeding or severe chewing and swallowing disorders (dysphagia) are also indications for the start of enteral nutrition (3). Enteral feeding should be started before malnutrition develops, as early treatment achieves better growth patterns (158). A PEG placement is recommended for enteral feeding planned for more than 2 months (138). However, the choice of enteral approach depends on GI motility. Gastric feeding is preferred, as it allows for boluses of food and can tolerate a larger volume and osmotic load than the small intestine, providing greater flexibility in the choice of formula type and feeding schedule (144).

Different strategies should be tailored to each patient depending on the extent of the motility disorder or feeding tolerance. First, continuous jejunal nutrition may recommended, especially in patients with delayed gastric emptying due to the risk of aspiration (18). For children with poor volume tolerance and high calorie needs, continuous overnight feeding combined with daytime boluses is recommended (3, 65, 138). Breast milk is the first choice for infants, while hypoosmotic and hydrolyzed formulas can be considered in children with dysmotility from 1 year of age (18). The addition of dietary fiber to enteral formulas has been shown to accelerate intestinal transit time, but the fiber content should reflect a normal diet (159). Although data are incomplete and conflicting, supplementation of medium-chain triglycerides to enteral formulas has been shown to improve symptoms such as chronic diarrhea, stunted growth, and poor appetite (160).

In cases of cow’s milk protein allergy, constipation can be a sign in children with neurological disorders. A cow’s milk protein exclusion diet for 12 months reduced constipation symptoms in a cohort study of 145 children with neurological disabilities (161). A whey-based formula may be attempted if gastric emptying is delayed (162). Formula containing whole casein protein slows gastric emptying compared to formula with whey protein. However, breast milk results in faster gastric emptying than different types of formula (163).

A polymeric enteral formula with standard energy density should be recommended (164), but children with increased energy needs and poor tolerance to large volumes of liquids may need formulas with high energy density containing fibers or supplementation with glucose polymers and/or long-chain triglycerides (140). For children with severely reduced mobility, a low-calorie maintenance formula with a low-fat content plus fiber and micronutrients is indicated (3). Enteral mixtures based on whey protein hydrolysates could be indicated in cases of slow gastric emptying or allergies to cow’s milk proteins (162). Enteral nutrition can be performed as intermittent boluses or continuously (165). Children with increased caloric requirements or poor volume tolerance may benefit from a combination of continuous nocturnal enteral nutrition combined with multiple boluses during the day (166).

Enteral nutrition with special formulas can be an effective intervention for neurologically impaired children with feeding difficulties (167). These special formulas can provide adequate nutrition while also addressing specific nutritional needs that may arise due to the child’s underlying condition. For example, natural formulas, which contain whole food ingredients, may be preferred over synthetic formulas in some cases as they can help maintain a diverse microbiota and gut health, which is crucial in individuals with gut dysmotility (168). In addition, the inclusion of fiber in enteral formulas has been shown to improve bowel function and reduce the incidence of constipation (169). Fiber is an indigestible carbohydrate that can promote the growth of beneficial gut bacteria and improve overall gut health (16). It can also provide bulk to stool, which can aid in bowel movements and prevent constipation (170).

Blenderized diets have been used in children with NI as an alternative to commercial enteral formulas, and may also be easier to digest and reduce the risk of constipation or other GI issues (154, 155). This type of diet can be tailored to meet the specific nutritional needs of the child and may be preferred by families who want to avoid processed foods or have concerns about the ingredients in commercial formulas (171). They could provide a wider range of nutrients and flavors than commercial formulas. Blenderized diets. Additionally, some parents report improvements in their child’s behavior, cognition, and energy levels after switching to a blenderized diet (172). Parents should work closely with a dietitian to develop a nutritionally balanced plan and should be aware of the potential risks of infection or nutrient imbalances if the diet is not properly prepared or stored. It is important to add that blenderized diets may not be appropriate for all children with NI and should only be used under the guidance of a healthcare professional. Factors such as medical history, feeding difficulties, and medication regimen should be taken into consideration when making this decision. A combination of blenderized feeding and commercial formulae may minimize symptoms of tube feeding whilst supporting growth (156).

Furthermore, selecting natural food options that are easily digestible and offer a variety of macronutrients and micronutrients can help ensure optimal nutrition (168). For neurologically impaired children with PEG, some natural food options that can be incorporated into their diet include: fruits and vegetables that are rich in vitamins, minerals, and antioxidants and can be easily blended into their feeding regimen; whole grains such as rice, oats, and quinoa, which provide carbohydrates, fiber, and essential nutrients; lean protein sources such as chicken, fish, and eggs that provide essential amino acids; and healthy fats such as avocado, nuts, and seeds that are rich in omega-3 fatty acids. Dairy products such as milk, yogurt, and cheeses are also good sources of calcium for bone health (166, 173, 174). Formulas containing natural foods have been shown to enhance tolerance and feeding outcomes, improve family participation, and increase compliance during mealtimes (175). In all cases, it is essential to consult a registered dietitian to develop an individualized nutrition plan for all patients with NI who are receiving enteral nutrition.

Special formulas can also be tailored to meet the specific nutritional needs of individual patients, particularly in the first years of life (176). For example, children with spasticity and dystonia may benefit from formulas with a higher protein content to support muscle growth and maintenance (177). Similarly, children with malabsorption may require peptide-based enteral formulas to promote absorption of essential nutrients (178). It is important to note that the use of special formulas should be guided by a healthcare professional and tailored to meet the individual needs of each patient. A registered dietitian can work with families and healthcare teams to develop individualized nutrition plans that address the specific needs of each patient.

### 6.4. Parenteral nutrition

In neurologically impaired children who have contraindications for enteral nutrition, or in those with severe gastrointestinal dysmotility who cannot tolerate enteral nutrition, parenteral nutrition may be considered (144). This type of nutrition, especially in the long term, is associated more with degenerative pathologies, and therefore more with neurogenetic/neurometabolic ones. Patients with spasticity and dystonia are at a higher risk of enteral feeding-induced dystonia, which can present with symptoms such as sweating, pallor, inconsolable crying, and convulsions (173). Interruption of enteral nutrition typically leads to an almost immediate response, indicating anterior gut dysmotility. The sympathetic motor fibers that innervate the anterior gut are controlled by higher centers located in the spinal cord and brainstem. Therefore, it is more likely for intestinal dysmotility to occur in central neurological disorders than in spinal lesions. Further research is needed to determine whether the neural modulation of gut motility occurs through direct effects of neurotransmitters or neuroendocrine mechanisms. In cases where enteral feeding-induced dystonia persists, total parenteral nutrition may be considered, but only after all enteral feeding options, medication intake, and pain control have been explored (179). This is an ethical approach to ensuring that parenteral nutrition is only used as a last medical choice.

## 7. Pharmacological treatments to increase the nutrition tolerance in children with GI dysmotility

Various drugs have been considered to improve oral nutrition and physiological peristalsis in children with intestinal dysmotility. The main mechanisms used include agents that stimulate smooth muscle contraction, also known as prokinetics, or, on the contrary, a delay in peristalsis, such as opioid receptor agonists (180). In addition, laxatives and anticholinergics can also be considered for constipation and diarrhea, respectively. Macrogol, also known as polyethylene glycol, is a water-soluble polymer which is commonly used in constipation management (181). Macrogol acts as a safe and effective osmotic laxative, increasing the water content of the stool and facilitating its movement through the bowel. It works by retaining water in the lumen of the intestine and colon, thus softening the stool and promoting bowel movements. In some cases, macrogol may be given daily as part of a long-term bowel management plan (182).

Overall, macrogol is a safe and effective treatment option for constipation in neurologically impaired children on enteral nutrition. However, as with any medication, it is important to consult with a healthcare provider before starting or changing the use of macrogol.

Listed here frequently uses drugs approved in cases of GI dysmotility in pediatric age:

- Dopaminergic receptor D2 antagonists (e.g., domperidone) promote normal esophageal motility, gastroduodenal peristalsis, and consequently gastric emptying, and are commonly used in gastroparesis, functional dyspepsia and GER (180). Metoclopramide acts as both a dopamine and serotonin receptor antagonist (both D1 and D2) and has demonstrated the ability to increase muscle tone of the middle and lower third of the esophagus in addition to tone of the esophageal sphincter, favoring instead pyloric relaxation and duodenal peristalsis (183). Its indications are the same as domperidone, but its antiemetic action also makes it useful for postoperative nausea and vomiting. Because it can cross the blood–brain barrier, especially when used for long periods and in high doses, it can be responsible for the occurrence of extrapyramidal symptoms (dystonia, tremor, tardive dyskinesia), which are sometimes slowly reversible.
- Selective serotonergic agents (e.g., prucalopride (5HT4), ondansetron (5HT3)) can instead be used in the treatment of gastroparesis and chronic pseudo-intestinal obstruction thanks to their activity in increasing motility and intestinal transit, thereby improving both the colonic and as well as the overall GI transit time can be accelerated (184, 185). In addition, ondansetron is effective in reducing episodes of vomiting and promoting oral rehydration, avoiding the use of IV hydration and hospitalization (186). It has shown a higher safety and efficacy profile than other antiemetic groups.
- Agonists of the motilin receptors, and specifically macrolides, have shown a promotility-promoting function at the GI level and are in fact used for this purpose. A recent meta-analysis collected the evidence for oral macrolide intake in promoting tolerance to enteral feeding in low-weight preterm infants who generally have problems with GI motility (187). Despite some limitations, the use of erythromycin for prophylactic purposes has shown a significant effect in promoting complete enteral nutrition in the newborn. In addition, their use has shown positive effects on the duration of parenteral nutrition and the overall length of hospital stay, and this effect does not seems to be dose-dependent. No significant morbidity was observed and indeed a lower incidence of cholestasis and necrotizing enterocolitis was observed in the treated subjects. However, doses and timing of administration have yet to be defined. Subsequently, acute therapy based on the use of macrolides (especially erythromycin and clarithromycin) may prove useful to promote tolerance in patients with various GI motility disorders, but further studies are needed to determine their dosage, timing, and safety profile.
- Inhibitors of acetylcholinesterase (e.g., neostigmine, pyridostigmine) belong to the category of acetylcholinesterase inhibitors. These drugs work by increasing the concentration of acetylcholine, a neurotransmitter that plays a key role in regulating gastrointestinal motility. By inhibiting acetylcholinesterase, these drugs can increase the effectiveness of acetylcholine in stimulating smooth muscle contractions and improving gut motility. Studies have shown that pyridostigmine and neostigmine can improve symptoms of gut dysmotility in children with conditions such as acute intestinal pseudo-obstruction and gastroparesis (188–190). However, further research is needed to determine the optimal dosing and long-term safety of these drugs in pediatric patients.
- Botulinum toxin, which acts on the release of acetylcholine from presynaptic cholinergic nerves and causes transient chemical denervation, has been used off-label in both adults and children for the treatment of conditions associated with hypertension and spasticity, esophageal achalasia, and cricopharyngeal spasm (191). Injecting botulinum toxin into the pylorus has been shown to speed up gastric emptying in severe conditions that have not responded to other treatments.

Table 1 reports prokinetic drugs and their relative dosages that can be used in cases of GI dysmotility.

**Table 1:** Prokinetic drugs used for GI dysmotility disorders.

Active principles	Mechanism of action	Pediatric dosage
Domperidone (180)*	Receptor antagonist of dopamine (D2)	10 mg up to three times a day
Metoclopramide (192)	Receptor antagonist of dopamine (D1 and D2) and serotonin (5HT3 and 5HT4)	0.1–0.15 mg/kg/dose up to 3 times a day
Neostigmine (188, 193)	Synthetic inhibitor, reversible, of acetylcholinesteras	0.01–0.05 mg/kg - off label
Pyridostigmine (190, 194)	Long-acting inhibitor action of acetylcholinesterase	0.25–2 mg/kg/die
Erythromycin (195)	Motilin agonist	3–5 mg/kg/dose
Prucalopride (184)	Selective agonist of serotonin 5HT4 receptor	0.03 mg/kg - off label
Ondansetron (196)	Selective antagonist of serotonin 5HT3 receptor	0.15 mg/kg iv (max 8 mg) or 2–8 mg by mouth up to three times a day
Cyproheptadine (197)	Antagonist of serotonin, histamine H1 and muscarinic receptors	0.19 mg/kg/day - off-label
Amoxicillin/clavulanic acid (198)	B-lattamic antibiotic drug	10 mg/kg twice daily - off-label
Botulinum toxin (191)	Neuromuscular blocker of acetylcholine release	Injection into external anal sphincter, dosage unknown
Baclofen (199)	Selective agonist of gamma-aminobutyric acid B (GABA-B) receptor	0.5–0.7 mg/kg once a day for 1 week

* Approved for children aged >12 years, weight ≥35 kg.

## 8. Conclusion

Patients with severe NI and neurometabolic disorders often have GI involvement that requires appropriate management. The underlying mechanisms leading to dysmotility are diverse, and the clinical manifestations are often nonspecific. It is crucial to distinguish neurometabolic disorders from other more common pediatric neurological injuries. Early diagnosis and appropriate therapy can reduce the progression to long-term neurological and GI sequelae.

Oral feeding, when safe and in the absence of the risk of ingestion or severe dysphagia, should always be encouraged to ensure adequate growth. When oral nutrition is insufficient or dangerous, it is necessary to switch to enteral nutrition before the onset of possible malnutrition. The ESPGHAN guidelines recommend gastrostomy as the best solution for long-term enteral nutrition in children with neurological disabilities, as it ensures the safest adequate caloric and fluid intake and the lowest rate of complications. Parenteral nutrition should be considered only after all enteral feeding options have been evaluated.

The role of the gut microbiota in health and disease is an area of increasing interest, particularly in relation to complex disorders such as NI and gastrointestinal dysmotility. Therefore, it is crucial to incorporate nutritional strategies that promote the growth of beneficial bacteria in the gut microbiota, such as prebiotics, probiotics, and fiber-rich foods, into the management of children with neurological impairment and gut dysmotility. Fermentable fibers, such as those found in fruits, vegetables, and whole grains, serve as a substrate for the growth of beneficial bacteria and can promote microbial diversity. These strategies may help to restore the gut microbiota’s diversity and promote a healthy gut-brain axis, leading to improvements in neurological symptoms and overall health outcomes.

Patients suffering from CP could benefit from the provision of fibers and prokinetics. In children suffering from dystrophies or mitochondrial diseases, the slowed gastric emptying time must be considered, and fiber overload can lead to abdominal distension and worsening of symptoms. Currently, there are no studies comparing different phenotypes of neurological patients. This could be a starting point for future research to define general problems of the neurological patient, stratify them according to etiological groups, and determine experimentally which treatment is the most appropriate approach to treating each disorder.

## Author contributions

EV and AC: conceptualization. LS and AC: methodology. CR, FG, and GZ: validation. AC, LS, AG, and EV: investigation. AC, LS, and AG: writing – original draft preparation. CR, EV, and FG: writing – review and editing. GZ and EV: supervision. All authors contributed to the article and approved the submitted version.

## Conflict of interest

The authors declare that the research was conducted in the absence of any commercial or financial relationships that could be construed as a potential conflict of interest.

## Publisher’s note

All claims expressed in this article are solely those of the authors and do not necessarily represent those of their affiliated organizations, or those of the publisher, the editors and the reviewers. Any product that may be evaluated in this article, or claim that may be made by its manufacturer, is not guaranteed or endorsed by the publisher.
